# A Novel Glycated Hemoglobin A1c-Lowering Traditional Chinese Medicinal Formula, Identified by Translational Medicine Study

**DOI:** 10.1371/journal.pone.0104650

**Published:** 2014-08-18

**Authors:** Hsin-Yi Lo, Chien-Yun Hsiang, Tsai-Chung Li, Chia-Cheng Li, Hui-Chi Huang, Jaw-Chyun Chen, Tin-Yun Ho

**Affiliations:** 1 Graduate Institute of Chinese Medicine, China Medical University, Taichung, Taiwan; 2 Department of Microbiology, China Medical University, Taichung, Taiwan; 3 Graduate Institute of Biostatistics, China Medical University, Taichung, Taiwan; 4 Graduate Institute of Cancer Biology, China Medical University, Taichung, Taiwan; 5 Department of Chinese Pharmaceutical Sciences and Chinese Medicine Resources, China Medical University, Taichung, Taiwan; 6 Department of Medicinal Botany and Healthcare, Da-Yeh University, Changhua, Taiwan; Massachusetts General Hospital, United States of America

## Abstract

Diabetes is a chronic metabolic disorder that has a significant impact on the health care system. The reduction of glycated hemoglobin A1c is highly associated with the improvements of glycemic control and diabetic complications. In this study, we identified a traditional Chinese medicinal formula with a HbA1c-lowering potential from clinical evidences. By surveying 9,973 diabetic patients enrolled in Taiwan Diabetic Care Management Program, we found that Chu-Yeh-Shih-Kao-Tang (CYSKT) significantly reduced HbA1c values in diabetic patients. CYSKT reduced the levels of HbA1c and fasting blood glucose, and stimulated the blood glucose clearance in type 2 diabetic mice. CYSKT affected the expressions of genes associated with insulin signaling pathway, increased the amount of phosphorylated insulin receptor in cells and tissues, and stimulated the translocation of glucose transporter 4. Moreover, CYSKT affected the expressions of genes related to diabetic complications, improved the levels of renal function indexes, and increased the survival rate of diabetic mice. In conclusion, this was a translational medicine study that applied a “bedside-to-bench” approach to identify a novel HbA1c-lowering formula. Our findings suggested that oral administration of CYSKT affected insulin signaling pathway, decreased HbA1c and blood glucose levels, and consequently reduced mortality rate in type 2 diabetic mice.

## Introduction

Diabetes is a major global health concern with a significant rise in prevalence. The diabetic population worldwide is increased dramatically from 153 million in 1980 to 347 million in 2008 [1]. In addition to prevalence, the economic cost for diabetes is also increased. The National Diabetes Information Clearinghouse estimates that diabetes costs $132 billion in United States in 2002 and the cost exceeds $245 billion in 2012 [2]. Therefore, diabetes has a significant impact on the health care system.

Based on the pathogenesis, diabetes is classified into two major categories: type 1 and type 2. Approximately 90–95% of the diabetic cases is type 2 diabetes. Moreover, increasing obesity and reduced activity levels lead to the rapid increased prevalence of type 2 diabetes. Type 2 diabetes displays the phenotype of hyperglycemia which results from insulin resistance and impaired insulin secretion. In addition to hyperglycemia, diabetes leads to both microvascular complications, such as retinopathy, nephropathy and neuropathy, and macrovascular complications, such as myocardial infarction and stroke [3]–[5].

It has been proved that improvement of glycemic control reduces or slows the progression of diabetic complications. For examples, the Diabetes Control and Complications Trial [6] proves that improvement of glycemic control reduces and prevents many of the early diabetic complications, such as retinopathy, microalbuminuria, nephropathy, and neuropathy. In addition, their results predict that individuals with the intensive diabetes management would gain an additional 5.1 years of life expectancy. Glycated hemoglobin A1c (HbA1c) is considered as the gold standard for monitoring glycemic control. A higher amount of HbA1c level indicates a poorer control of blood glucose and a higher prevalence of diabetic complications. Moreover, a regular assessment of HbA1c level in diabetic patients allows the adjustment of patients' drugs or dosages [7], [8]. With the increasing use of combination therapy with synthetic drugs and Chinese medicinal formulae in diabetic patients in Taiwan, we proposed that some Chinese medicinal formulae might exhibit HbA1c-lowering potentials in clinics. Therefore, we applied a translational medicine study to prove our speculation. By surveying the clinical evidences from diabetic patients enrolled in Diabetes Care Management Program, we found that Chu-Yeh-Shih-Kao-Tang (CYSKT) exhibited a HbA1c-lowering potential. The effects of CYSKT on the regulation of HbA1c and blood glucose in type 2 diabetic mice were further elucidated in this study.

## Materials and Methods

### Materials

Chinese medicinal formula CYSKT was purchased from the GMP pharmaceutical company (Sun Ten Pharmaceutical Co., Taipei, Taiwan). CYSKT was composed of seven ingredients: bamboo leaves (5.1%), gypsum (41%), pinellia rhizome (10.3%), ginseng root (7.7%), licorice root (5.1%), rice (15.4%), and ophiopogon tuber (15.4%). The ethanolic extract of CYSKT was analyzed by high-performance liquid chromatography using glycyrrhizin (Sigma, St. Louis, MO, USA) as a reference standard (Figure S1). The details of high-performance liquid chromatography is described in Text S1. Each gram of freeze-dried extract of CYSKT contained 90 mg of glycyrrhizin.

### Study Participants

The clinical data analyzed in this study were obtained from Diabetes Care Management Program, which was launched by Taiwan National Health Insurance Administration. Study participants were patients diagnosed as diabetes with International Classification of Diseases, Nine Revision, Clinical Modification code ICD-9-CM250. A total of 64,878 diabetic patients was enrolled in Diabetes Care Management Program between 2001 and 2004. The selection criteria for this study was that patients had follow-up records for at least one year. A total of 9,973 participants who met the aforementioned criteria was selected in this study. We applied a retrospectic cohort study. The independent variables were patient's age, gender, family, history, health behavior, complications, comorbidities, and Chinese medicinal formula usage. The dependent variable was the change of HbA1c value between baseline and one-year follow-up records of patients. This study was approved by Ethics Review Board of China Medical University Hospital (Permit No. DMR97-IRB-272). Patient records and information were anonymized and de-identified prior to analysis.

### Animal Experiments

BALB/c mice were obtained from National Laboratory Animal Center (Taipei, Taiwan). Mouse experiments were conducted under ethics approval from China Medical University Animal Care and Use Committee (Permit No. 101-188-N). Mice were maintained under a 12∶12 light-dark cycle with free access to water and food unless indicated.

Mice with type 2 diabetes were generated by a combination of high-fat diet-induced insulin resistances and low-dose streptozotocin-induced defects in insulin secretion [9], [10]. Briefly, 4-week-old male BALB/c mice were fed with high-fat diet (TestDiet, St. Louis, MO, USA), in which 60% of energy was from fat. Three weeks later, mice were intraperitoneally injected once with 100 mg/kg streptozotocin and fed with high-fat diet for another 3 weeks. At 10 weeks of age, mice were bled via tail veins after 4 h starvation and blood glucose levels were measured by a glucose oxidase method using a glucometer (ACCU-CHEK Advantage, Roche Diagnostics, Basel, Switzerland). High-fat diet- and streptozotocin-induced mice with fasting blood glucose levels ≥230 mg/dL were selected and then divided randomly into mock, CYSKT, TZD, and CYSKT/TZD groups. Normal mice were divided randomly into mock and CYSKT groups. Glucose tolerance test was performed as described previously [11]. Data were expressed as area under the curve (AUC). Serum renal function indexes, such as blood urea nitrogen (BUN) and creatine, were measured using an autoanalyzer (COBAS Mira Plus, Roche Diagnostics, Basel, Switzerland).

### Microarray Analysis

Type 2 diabetic mice were given orally with 200 mg/kg CYSKT for 30 consecutive days. Total RNAs were extracted from muscle tissues using RNeasy Mini kit (Qiagen, Valencia, CA, USA) and analyzed for RNA integrity by Agilent 2100 bioanalyzer (Agilent Technologies, Santa Clara, CA, USA). Microarray analysis was performed as described previously [12], [13]. Briefly, fluorescence-labeled RNA targets were hybridized to the Mouse Whole Genome OneArray (Phalanx Biotech Group, Hsinchu, Taiwan) and fluorescent signals on the array were scanned by an Axon 4000 scanner (Molecular Devices, Sunnyvale, CA, USA). The fluorescent intensity of each spot was analyzed by genepix 4.1 software (Molecular Devices, Sunnyvale, CA, USA) and normalized by R program in the limma package using quantile normalization. For pathway analysis, normalized data were analyzed using the “geneSetTest” function implemented in the limma package to detect groups of genes in Kyoto Encyclopedia of Genes and Genomes pathways (http://www.genome.jp/kegg/pathway.html). This function computes a *p*-value to test the hypothesis that the selected genes in the pathway tend to be differentially expressed. For diseases analysis, we built 735 disease-gene sets from genetic association database according to Medical Subject Headings (MeSH) terms (http://www.nlm.nih.gov/mesh/meshhome.html) [14]. We applied “geneSetTest” function to detect groups of genes in MeSH disease terms. This function computes a *p*-value to test the hypothesis that the selected genes tend to be upregulated or downregulated. Microarray data are MIAME compliant and raw data have been deposited in the Gene Expression Omnibus (Accession number: GSE53119). Number of replicate was three.

### Cell Culture and Insulin Receptor (IR)-Binding Assay

HepG2 cells were maintained in Dulbecco's modified Eagle's medium (DMEM) (Invitrogen, Carlsbad, CA, USA) supplemented with 10% fetal bovine serum (Hyclone, Logan, UT, USA). HepG2 cells were cultured in 25-cm^2^ flasks at 37°C. After a 24-h incubation, cells were treated with various amounts of CYSKT in DMEM, incubated at 37°C for 10 min, and then solubilized by lysis buffer (1% NP-40, 20 mM Tris-HCl, pH 8.0, 137 mM NaCl, 10% glycerol, 2 mM EDTA, 1 mM sodium orthovanadate, 10 µg/mL aprotinin, 10 µg/mL leupeptin). Cell lysates were centrifuged at 2,000×g for 5 min at 4°C, supernatants were collected, and protein concentrations in supernatants were quantified using a Bradford method (Bio-Rad, Hercules, CA, USA). For *in vivo* experiment, BALB/c mice were fasted for 18 h and then given orally with various amounts of CYSKT. One hour later, mice were sacrificed, and livers were collected and solubilized by lysis buffer. IR-binding assay was performed using Phospho-IR set (R&D Systems, Minneapolis, MN). The amount of phosphorylated IR was quantified by sandwich enzyme-linked immunosorbent assay (ELISA). The absorbance at 450 nm was measured in an ELISA plate reader.

### Immunohistochemical Staining (IHC)

Parafilm-embedded muscle tissues were cut into 5-µm sections, deparaffinized in xylene, and then rehydrated in graded alcohol. Endogenous peroxidase was quenched with 3% hydrogen peroxide in methanol and the nonspecific binding was blocked with 1% bovine serum albumin. Sections were incubated with rabbit polyclonal antibody against glucose transporter 4 (GLUT-4) (Chemicon, Temecula, CA, USA) at 1∶200 dilution overnight at 4°C and then incubated with biotinylated secondary antibody (Zymed Laboratories, South San Francisco, CA, USA) at room temperature for 20 min. Finally, slides were incubated with avidin-biotin complex reagent and stained with 3,3′-diaminobenzidine according to manufacturer's protocol (Histostain-Plus, Zymed Laboratories, South San Francisco, CA, USA). GLUT-4 positive areas were measured using Image-Pro Plus (Media Cybernetics, Bethesda, MD, USA) to quantify the expression of GLUT-4. The proportions of GLUT-4 area (%) were calculated as area occupied with brown color/area of whole field.

### Statistical Analysis

Differences in HbA1c values and CYSKT usage were assessed using χ^2^ test and further analyzed by multiple linear regression analysis and multiple logistic regression. All analyses were conducted using SAS statistical software version 9.1 (SAS Institute Inc., Cary, NC, USA). Data from cellular and animal experiments were presented as mean ±standard deviation (SD). Student's *t* test was used for comparisons between two experiments. Results were considered statistically significant if 2-tailed *p* values were less than 0.05.

## Results

### Characteristics of Study Participants

A total of 64,878 diabetic patients was enrolled in Diabetic Care Management Program between 2001 and 2004, and a total of 9,973 patients who had follow-up records for at least one year was selected in this study. Details of demographic distribution of participants are shown in Table 1. The average age of participants was 60.52±11.62 years old. The majority (96.72%) of study participants was type 2 diabetes. The average value of HbA1c among all participants was 8.11±1.86% (65±20.3 mmol/mol).

**Table 1:** Demographic information of participants in this study.

Variable	Population (*n* = 9,973)	
	Number	Percentage
Age intervals		
<40 y	389	3.90
40–50 y	1,299	13.03
50–60 y	2,603	26.10
60–70 y	3,297	33.06
>70 y	2,385	23.91
Type of diabetes		
Type 1	162	1.62
Type 2	9,646	96.72
Other	71	0.71
Loss	94	0.95
HbA1c value		
<7% (53 mmol/mol)	3,050	30.58
≥7% (53 mmol/mol)	6,844	68.63
Loss	79	0.79
History intervals		
0–10 y	7,139	71.58
>10 y	2,781	27.89
Loss	53	0.53

By analyzing the types of medical care of 9,973 patients, we found that 9,444 patients (94.7%) used synthetic drugs alone and 529 patients (5.3%) used both synthetic drugs and medicinal formulae for the treatment of diabetes. The top ten most commonly used Chinese medicinal formulae are shown in Table S1. We further analyzed the relationship between medicinal formula usage and HbA1c levels in type 2 diabetic patients who used both synthetic drugs and medicinal formulae. The HbA1c values of patients taking with CYSKT were significantly reduced in comparison with those taking without CYSKT by multiple linear regression analysis (1.1% vs. 0.14%, *p* = 0.0025). The odds ratio of reduction in HbA1c levels in patients taking with CYSKT was significantly higher (3.55-fold, *p* = 0.0036, 95% confidence intervals) than those taking without CYSKT by multiple logistic regression. Synthetic drugs used in patients taking with CYSKT and the duration of CYSKT administration are shown in Table S2 and Figure S2. These findings suggested that administration of CYSKT might reduce the HbA1c levels in diabetic patients. Therefore, the effects of CYSKT on the regulation of HbA1c and blood glucose levels were analyzed in type 2 diabetic mice.

### Effects of CYSKT on HbA1c and Blood Glucose Levels in Type 2 Diabetic Mice

**Figure 1:**
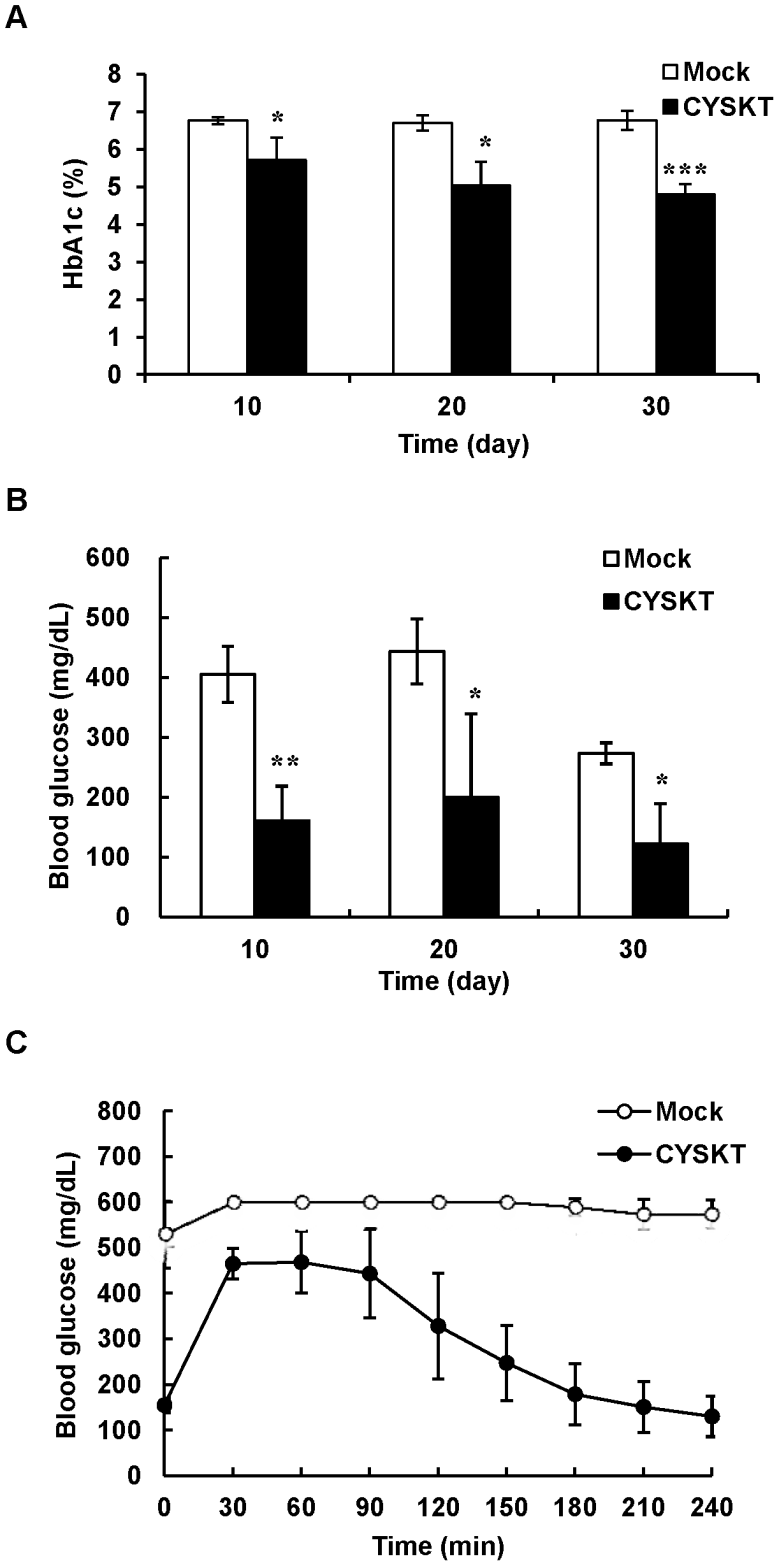
Effects of CYSKT on the levels of HbA1c and blood glucose in type 2 diabetic mice. Type 2 diabetic mice were administered orally 200 mg/kg CYSKT for 30 consecutive days. Blood samples were collected every 10 days and measured for HbA1c levels (A) and fasting blood glucose levels (B). Glucose tolerance test was performed on 30th day after CYSKT administration. Mice were fasted for 4 h, glucose (1 g/kg) was injected intraperitoneally, and the blood glucose levels at intervals were measured (C). Values are mean ± SD (*n* = 5). *p<0.05, **p<0.01, ***p<0.001, compared with mock.

**Figure 2:**
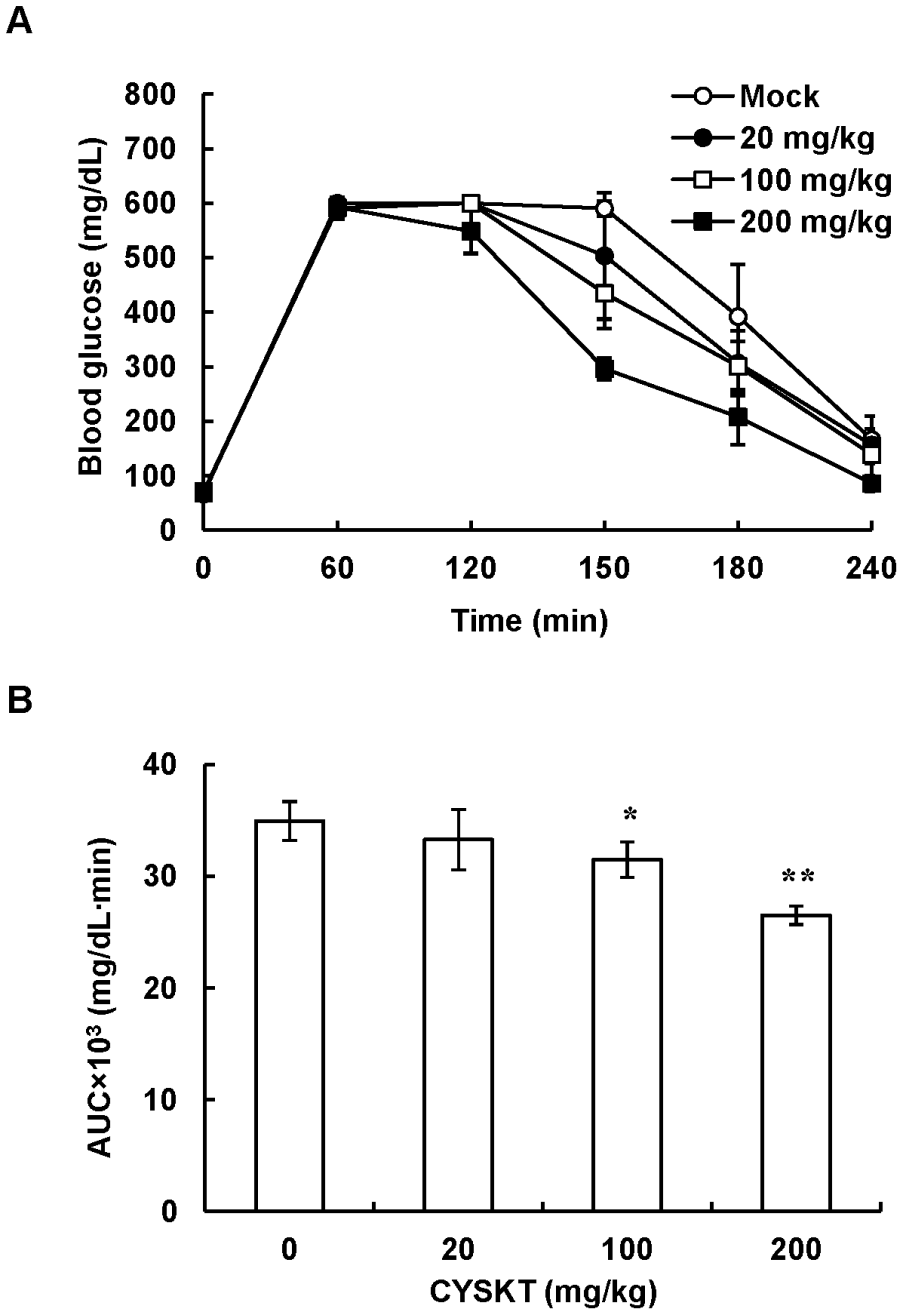
Effect of CYSKT on blood glucose levels in normal mice. Normal mice were administered orally various amounts of CYSKT. Glucose (4 g/kg) was injected intraperitoneally 15 min later, blood samples were collected at intervals, and blood glucose levels were measured by a glucometer (A). (B) AUC of glucose tolerance assay. Values are mean ± SD (*n* = 5). *p<0.05, **p<0.01, compared with mock.

Type 2 diabetic mice were given orally with 200 mg/kg CYSKT for 30 consecutive days and blood samples were collected every 10 days. As shown in Figure 1A, HbA1c levels of mock mice were approximately 7% (53 mmol/mol). CYSKT significantly lowered the HbA1c levels and the reduction displayed a time-dependent manner. The fasting blood glucose levels were also measured every 10 days and the glucose tolerance test was performed on the 30th day. As shown in Figure 1B, in comparison with mock, CYSKT significantly lowered the fasting blood glucose levels measured on the 10th, 20th, and 30th days. Glucose tolerance test showed that mock group displayed a very poor glucose clearance ability, while oral administration of CYSKT displayed a rapid clearance of glucose (Figure 1C). AUC values were also significantly decreased by CYSKT, in comparison with mock (36360±8104.4 mg/dL·min vs. 70687.5±711.7 mg/dL·min, *p* = 0.001865). In addition to diabetic mice, normal mice were given orally with various amounts (10, 100, and 200 mg/kg) of CYSKT. As shown in Figure 2, the fasting blood glucose level was approximately 70 mg/dL and the blood glucose concentration reached a maximal level at 60 min after glucose challenge. Mock group displayed a poor glucose clearance. Oral administration of CYSKT significantly stimulated the blood glucose clearance and the stimulation displayed a dose-dependent manner. These data indicated that CYSKT alone exhibited HbA1c-lowering and hypoglycemic effects in normal and type 2 diabetic mice.

### Analysis of HbA1c-Lowering and Hypoglycemic Mechanisms of CYSKT in Type 2 Diabetic Mice

To elucidate the hypoglycemic mechanism of CYSKT, we collected muscle tissues from type 2 diabetic mice given orally with 200 mg/kg CYSKT for 30 consecutive days, extracted RNA samples from muscle tissues, and performed microarray analysis. In a total of 29,922 transcripts, 283 transcripts with fold change ≥1.5 or ≤−1.5 were selected for pathway analysis. A total of 69 pathways was significantly regulated by CYSKT and the affected pathways involved in glucose and lipid metabolism are shown in Table 2. CYSKT significantly affected insulin, insulin-like growth factor, peroxisome proliferator-activated receptors (PPAR), free fatty acid, leptin, and adipocytokine signaling pathways. CYSKT also significantly regulated citrate cycle and fatty acid metabolism. These data suggested that CYSKT might regulate HbA1c and blood glucose levels via insulin signaling pathway.

**Table 2:** Pathways significantly regulated by CYSKT in type 2 diabetic mice*.

Pathway†	*p* value‡
Citrate cycle	1.45×10^−9^
Insulin signaling pathway	0.000117
Insulin-like growth factor signaling pathway	0.000142
PPAR signaling pathway	0.000311
Fatty acid metabolism	0.000747
Free fatty acid signaling pathway	0.001233
Fatty acid elongation in mitochondria	0.001549
Glycerolipid metabolism	0.008970
Leptin signaling pathway	0.018841
Adipocytokine signaling pathway	0.040090

*Type 2 diabetic mice were given orally with 200 mg/kg CYSKT for 30 consecutive days. Total RNAs were extracted from muscle tissues and analyzed by microarray.

† Pathways associated with glucose and lipid metabolism are shown.

‡ *p* values were calculated by geneSetTest function implemented in the limma package.

**Figure 3:**
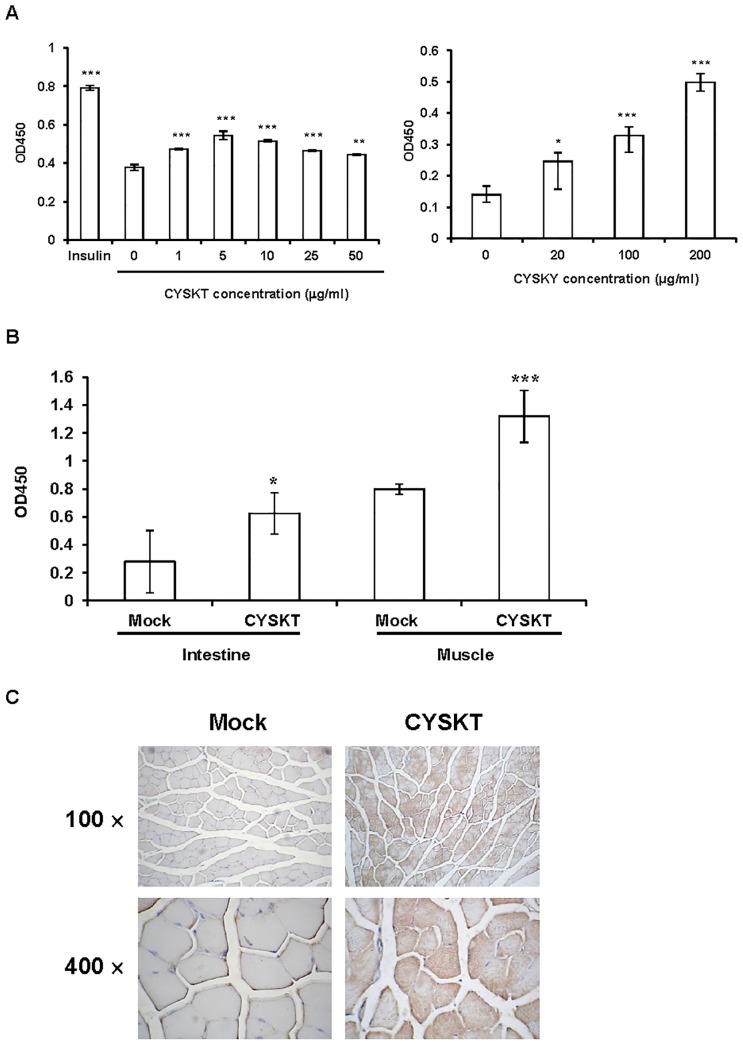
Effects of CYSKT on IR phosphorylation and GLUT-4 translocation. (A) Phospho-IR ELISA. Left panel: HepG2 cells were treated with 0.5 µM insulin or various amounts of CYSKT. Ten minutes later, cellular proteins were collected and the levels of phosphorylated IR were measured by ELISA. Values are mean ± SD (*n* = 6). Right panel: BALC/c mice were given orally with various amounts of CYSKT. One hour later, mice were sacrificed, and livers were collected for analysis. Values are mean ± SD (*n* = 6). *p<0.05, ***p<0.001, compared with mock. (B) Phospho-IR ELISA. Type 2 diabetic mice were administered orally 200 mg/kg CYSKT for 30 days. Proteins were extracted from intestines and muscles, and the levels of phosphorylated IR were measured by ELISA. Values are mean ± SD (*n* = 5). *p<0.05, **p<0.01, ***p<0.001, compared with mock. (C) IHC. Type 2 diabetic mice were administered orally 200 mg/kg CYSKT for 30 consecutive days. Muscle tissues were collected and the sections were stained by IHC using antibody against GLUT-4 (100× and 400× magnification). Photos are representative images (*n* = 5).

We further analyzed effects of CYSKT on IR activation and GLUT-4 translocation. We treated HepG2 cells, which have been known to express IR on cell membranes, with CYSKT and measured the amounts of phosphorylated IR by ELISA. As shown in Figure 3A (left panel), insulin significantly increased the levels of phosphorylated IR. CYSKT also stimulated the phosphorylation of IR and the maximal induction was achieved by 5 µg/mL CYSKT. In addition to HepG2 cells, we also analyzed the amounts of phosphorylated IR in livers of normal mice. As shown in Figure 3A (right panel), the amounts of phospho-IR in livers were significantly increased by CYSKT in a dose-dependent manner. These data suggested that CYSKT might interact physically with IR and stimulate the phosphorylation of IR. Buts et al [15] indicate that enterocytes of rodents express IR. Because CYSKT was administered orally and we proposed that CYSKT might activate IR signaling pathway, we chose small intestinet for the IR phosphorylation experiment. In addition, oral administration of 200 mg/kg CYSKT for 30 days also significantly increased the levels of phosphorylated IR in intestines and muscles of type 2 diabetic mice (Figure 3B). These findings suggested that CYSKT activated insulin signaling pathway by stimulating the phosphorylation of IR. Following IR phosphorylation, GLUT-4, an insulin-dependent glucose transporter, is translocated into cell membrane and uptakes blood glucose into cells. We therefore analyzed the translocation of GLUT-4 in muscle by IHC. Figure 3C shows that, in comparison with mock, there were many immuno-reactive cells in CYSKT-treated muscle tissues. The proportions of GLUT-4 area (%) were 27.2±4.8% in mock group and 84.9±9.7% in CYSKT group (*p*<0.001). These data suggested that CYSKT exhibited HbA1c-lowering and hypoglycemic effects by activating the phosphorylation of IR and enhancing the translocation of GLUT-4 to the cell membrane.

### Effects of CYSKT on Diabetic Complications and Survival Rate in Type 2 Diabetic Mice

**Figure 4:**
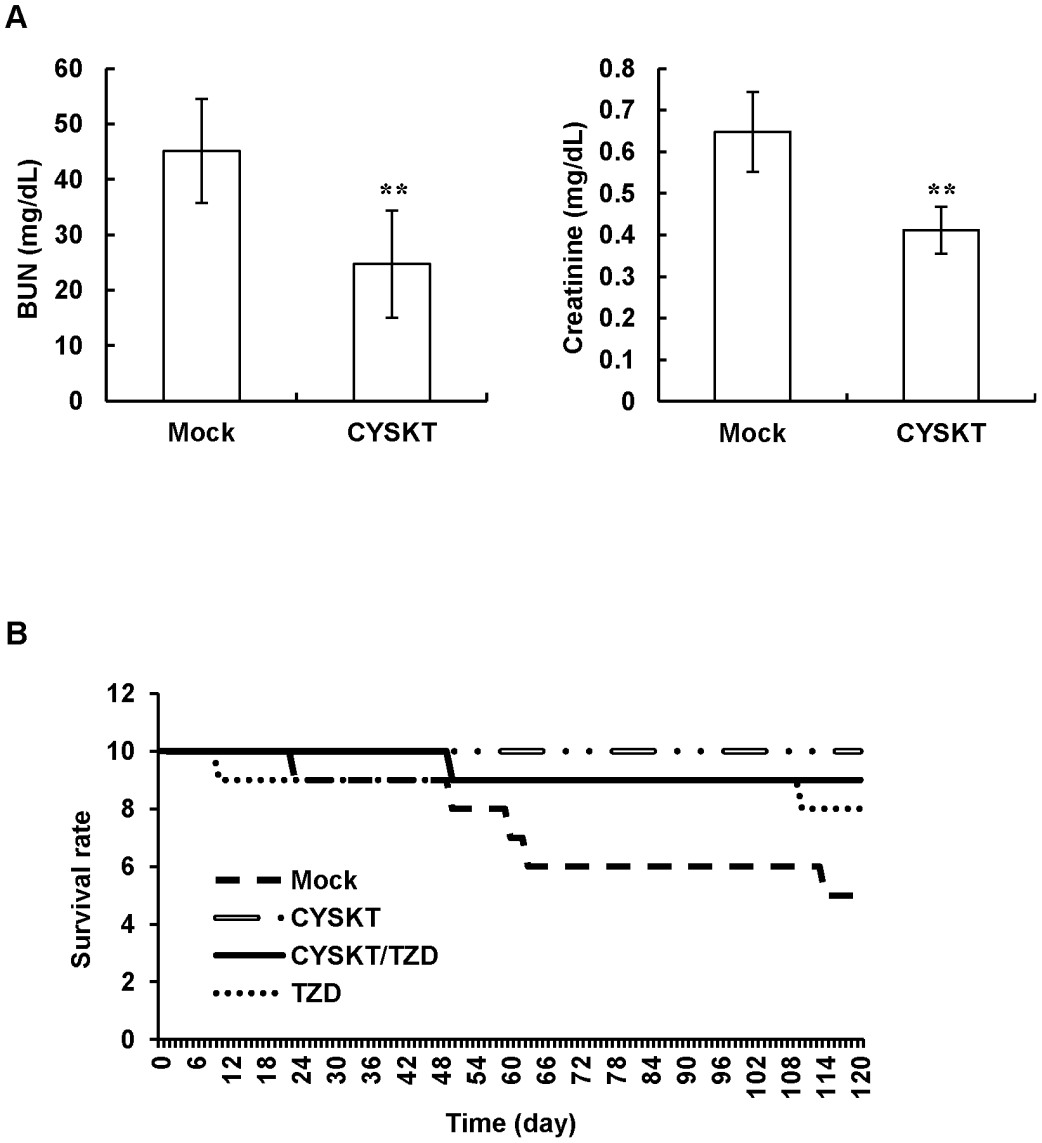
Effects of CYSKT on renal function indexes and survival rate in type 2 diabetic mice. (A) CYSKT (200 mg/kg) was orally given to type 2 diabetic mice for 30 consecutive days. The levels of BUN and creatinine in sera were measured by an autoanalyzer. Values are mean ± SD (*n* = 5). **p<0.01, compared with mock. (B) Long-term survival rate. Type 2 diabetic mice were administered orally with 200 mg/kg CYSKT and/or 20 mg/kg TZD for 120 consecutive days. The number of death was observed daily.

The reduction of HbA1c is highly associated with the improvement of glycemic control and diabetic complications. The effects of CYSKT on diabetic complications were further analyzed by gene expression profiling, renal function indexes, and survival rate. Type 2 diabetic mice were given orally with 200 mg/kg CYSKT for 30 consecutive days, and RNA samples from muscle and serum samples were collected for microarray and serum biochemical analyzes, respectively. By analyzing the similarity between CYSKT-affected genes and disease-affected genes, we found that 55 terms were significantly affected by CYSKT among 735 tested MeSH disease terms. CYSKT affected genes associated with diabetes (Table 3). It also affected gene sets involved in diabetic complications, such as retinopathy, nephropathy, neuropathy, and cardiovascular disorders. These data indicated that CYSKT might affect the progression of diabetic complications. We further analyzed the levels of BUN and creatinine in sera. CYSKT significantly decreased the levels of BUN and creatinine (Figure 4A), suggesting that CYSKT might improve the diabetic renal complications in type 2 diabetic mice. It is known that the improvement of diabetic complications prolongs the lifespan of diabetic patients [4]. Therefore, we administered type 2 diabetic mice with 200 mg/kg CYSKT for 120 days and observed the number of death daily. As shown in Figure 4B, the survival rate of mock and CYSKT groups were 5/10 and 10/10, respectively, at the end of experiments. In addition, the survival rate of CYSKT treatment was similar to those of thiazolidinedione (TZD) treatment (8/10) and TZD/CYSKT combinatory therapy (9/10). Moreover, in comparison with mock, administration of CYSKT in CYSKT and CYSKT/TZD groups decreased the levels of HbA1c (Figure S3). These data suggested that CYSKT regulated HbA1c and blood glucose values, improved diabetic complications, and consequently decreased the mortality rate of type 2 diabetic mice.

**Table 3:** Gene-expression connection of CYSKT treatments with disease states*.

MeSH disease term	*p* value†
Diabetes mellitus, type 2	0.0012
Uremia	0.0035
Macular degeneration	0.0045
Diabetic retinopathy	0.0079
Diabetic angiopathies	0.0092
Myocardial ischemia	0.0126
Coronary vasospasm	0.0204
Heart diseases	0.0375

*Type 2 diabetic mice were given orally with 200 mg/kg CYSKT for 30 consecutive days. Total RNAs were extracted from muscle tissues and analyzed by microarray.

† *p* values were calculated by geneSetTest function implemented in the limma package.

## Discussion

In this study, we performed a translational medicine research on a traditional Chinese medicinal formula CYSKT. By surveying the medical usage of diabetic patients enrolled in Diabetes Care Management Program and analyzing the association between medical usage and HbA1c values, we found that CYSKT exhibited a HbA1c-lowering potential in diabetic patients. We chose the changes of HbA1c values instead of fasting blood glucose levels as the dependent variable because HbA1c is considered as the gold standard for monitoring glycemic control [7], [8]. The formation of HbA1c mainly depends on the interaction between blood glucose concentrations and life span of red blood cells. Therefore, HbA1c value represents the average blood glucose level of a patient in past 2 to 3 months [16], [17]. In addition, clinical studies indicate that reduction in HbA1c values directly reflects the improvement of diabetic complications. For examples, a 37% reduction in combined microvascular complications, a 21% reduction in diabetes-related deaths, and a 14% decrease in combined fatal and nonfatal myocardial infarction are noted for every 1% reduction in HbA1c value. Moreover, the strict glycemic control and the reduction of HbA1c to 7% (53 mmol/mol) or less lead to a decreased incidence of microvascular and macrovascular complications [18]–[20].

The increasing use of complementary and alternative medicine among the general public has been noticed [21]. By surveying the medical choice of patients, we also found that, although there are several synthetic drugs available for diabetes, there are 5.3% of study participants used both synthetic drugs and medicinal formulae for the treatment of diabetes. The use of medicinal formulae or herbs has the advantage that they do not cause significant side effects, in comparison with synthetic drugs. For examples, metformin may increase the risk of lactic acidosis and gastrointestinal side effects [22], while sulfonylurea treatment may result in a significant hypoglycemia or weight gain [23]. Chinese medicinal herbs have been used in the clinics for the treatment of diabetes for years. Yeh et al [24] review the clinical researches and find that *Coccinia indica* and American *ginseng* decrease the levels of fasting blood glucose and HbA1c in patients [25], [26]. *Gymnema sylvestre*, *Aloe vera*, and *Momordica charantia* also display the clinical effectiveness in patients in nonrandomized trials or short-term metabolic trials [27]–[29]. Therefore, we tried to figure out the Chinese medicinal formulae with HbA1c-lowering potential from clinical data, and our findings suggested that CYSKT was a candidate because diabetic patients taking with CYSKT showed lower HbA1c levels than those taking without CYSKT.

CYSKT is a traditional Chinese medicinal formula that has been used for the treatment of respiratory diseases and diabetes in China for years. It is comprised of seven ingredients. The major ingredient of CYSKT is gypsum, followed by ophiopogon tuber, rice, pinellia rhizome, ginseng root, bamboo leaves, and licorice root. So far, several ingredients of CYSKT have showed the hypoglycemic effects in diabetic animals or patients. For examples, the polysaccharide from the tuber of *Ophiopogon japonicus* improves glucose tolerance and impaired insulin secretion via phosphatidylinositide 3-kinase (PI3K)/Akt pathway in diabetic animals [30]. It also protects diabetes-caused hepatol and renal injuries via antioxidant ability in rats [31]. Ginseng, the root of *Panax ginseng*, alleviates the hyperglycemia and insulin resistance of type 2 diabetic animals [32]. The anti-diabetic effects of ginseng have also been reported in the clinical trials [33]. Licorice root, the radix of *Glycyrrhiza uralensis*, enhances the insulin-stimulated glucose uptake through PPAR-γ activation in 3T3-L1 cells and improves the glucose tolerance in diabetic animals [34]. In this study, we found that CYSKT steadily decreased the levels of blood glucose and HbA1c in type 2 diabetic mice. The ingredients, such ophiopogon tuber, ginseng root and licorice root, might be responsible for the hypoglycemic effect of CYSKT.

Type 2 diabetes is characterized by both insulin resistance and impaired insulin secretion. Insulin secretagogues like sulfonylureas are used to stimulate insulin secretion by interacting with the ATP-sensitive potassium channel on the pancreatic beta cells. TZDs bind to PPAR-γ nuclear receptor and improve the insulin resistance [35]. In this study, we suggested that CYSKT ameliorated the hypoglycemic status of type 2 diabetic mice by activating the autophosphorylation of IR. IR is a transmembrane protein which exhibits tyrosine kinase activity. Upon binding of insulin to the extracellular domain of IR, the tyrosine kinase is activated and then proceeded the autophosphorylation of IR and cellular proteins, which leads to the activation of PI3K and translocation of GLUT-4 [36]. CYSKT increased the amount of phosphorylated IR and stimulated the translocation of GLUT-4 to cell membrane suggested that CYSKT might bind to IR, activate the phosphorylation of IR, and stimulate the down-stream signaling pathway. In our previous study, we have identified that the protein component from *Momordica charantia* binds to IR, activates the insulin signaling pathway, and lowers the blood glucose levels in diabetic mice [11]. Zhao et al [37] have also found that Chinese medicinal herbs containing berberine exhibit sustained antidiabetic effects via altering hepatic gene expression. We suggested that the chemical or protein constituents of CYSKT might bind to IR and then improve the insulin resistance in type 2 diabetic mice.

In conclusion, we applied a translational medicine study to figure out that CYSKT exhibited the HbA1c-lowering potential by surveying 9,973 patients enrolled in Diabetes Care Management Program. Our data showed that CYSKT indeed was a HbA1c-lowering formula that steadily decreased the levels of HbA1c and blood glucose by activating the insulin signaling pathway and enhancing the translocation of GLUT-4 to the cell membrane, and consequently reduced the mortality in type 2 diabetic mice.

## Supporting Information

Figure S1 **HPLC chromatograph of CYSKT using glycyrrhizin as a reference standard.** (A) The chromatogram of ethanolic extract of CYSKT. (B) The chromatogram of glycyrrhizin standard. The retention time of glycyrrhizin was 47.3 min. Arrow indicate the peaks representing glycyrrhizin.
(PDF)

Figure S2 **The duration of CYSKT administration and HbA1c measurement in diabetic patients.** Rectangle represents the duration of CYSKT administration. Triangle represents the time of HbA1c measurement. The number below each triangle represents the HbA1c value (%).
(PDF)

Figure S3 **Effects of CYSKT on the HbA1c levels in type 2 diabetic mice.** Type 2 diabetic mice were administered orally with 200 mg/kg CYSKT and/or 20 mg/kg TZD for 120 consecutive days. Sixty days after CYSKT administration, blood samples were collected every 30 days and measured for HbA1c levels. Values are mean ± SD (*n* = 5).
(PDF)

Table S1 **The top ten commonly used Chinese medicinal formulae in diabetic patients.**
(PDF)

Table S2 **List of synthetic drugs used in patients who also administered CYSKT.**
(PDF)

Text S1 **Materials and Methods** **for Figure S1.**
(DOCX)
