# Supplementary figures and images for: A Novel Glycated Hemoglobin A1c-Lowering Traditional Chinese Medicinal Formula, Identified by Translational Medicine Study
Source: PLoS One. 2014 Aug 18;9(8):e104650. doi: 10.1371/journal.pone.0104650 (PMC4136774; doi:10.1371/journal.pone.0104650)

**Figure S1**

**(A)**

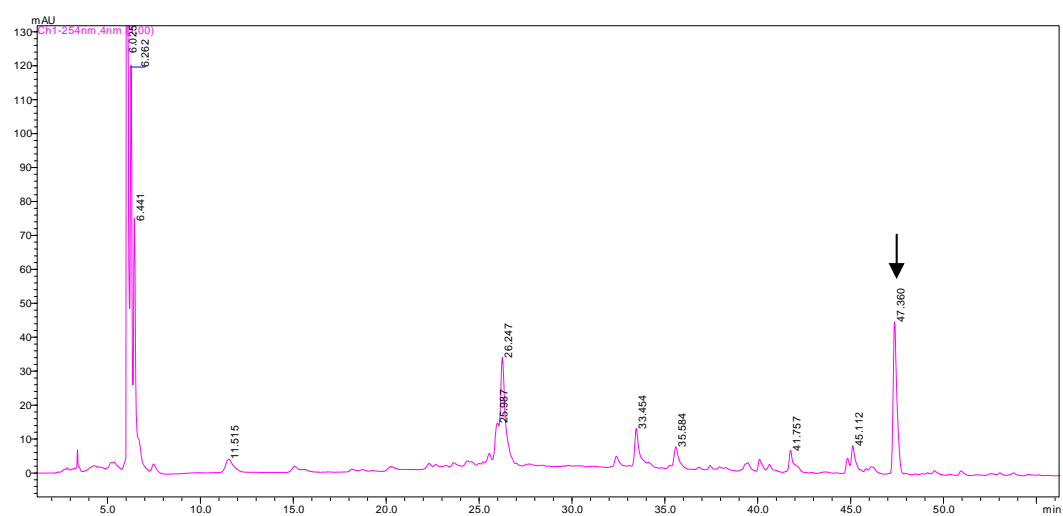

**(B)**

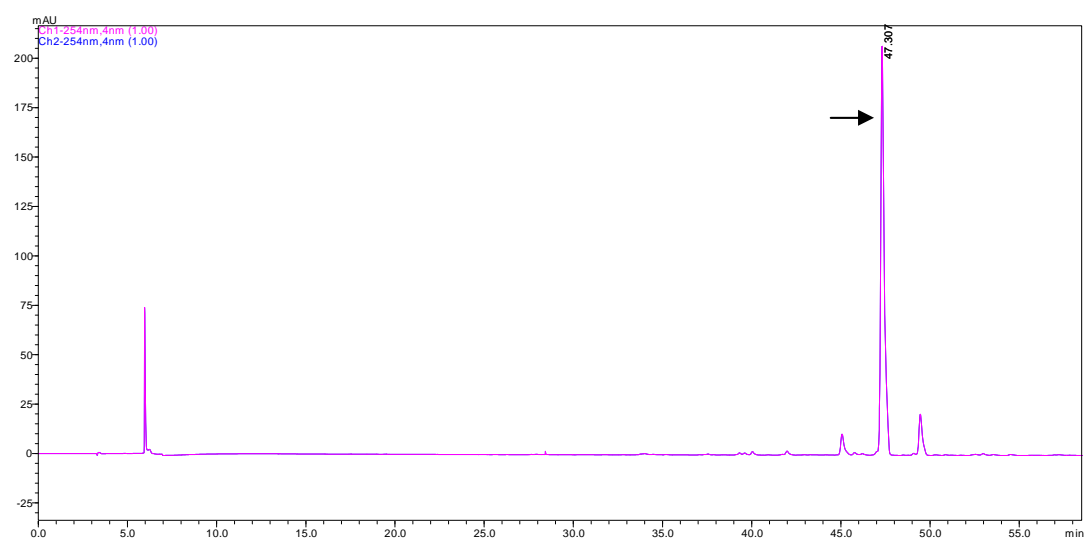

Supplement: Figure S1 — HPLC chromatograph of CYSKT using glycyrrhizin as a reference standard. (A) The chromatogram of ethanolic extract of CYSKT. (B) The chromatogram of glycyrrhizin standard. The retention time of glycyrrhizin was 47.3 min. Arrow indicate the peaks representing glycyrrhizin. (PDF) [file pone.0104650.s001.pdf]

Figure S2

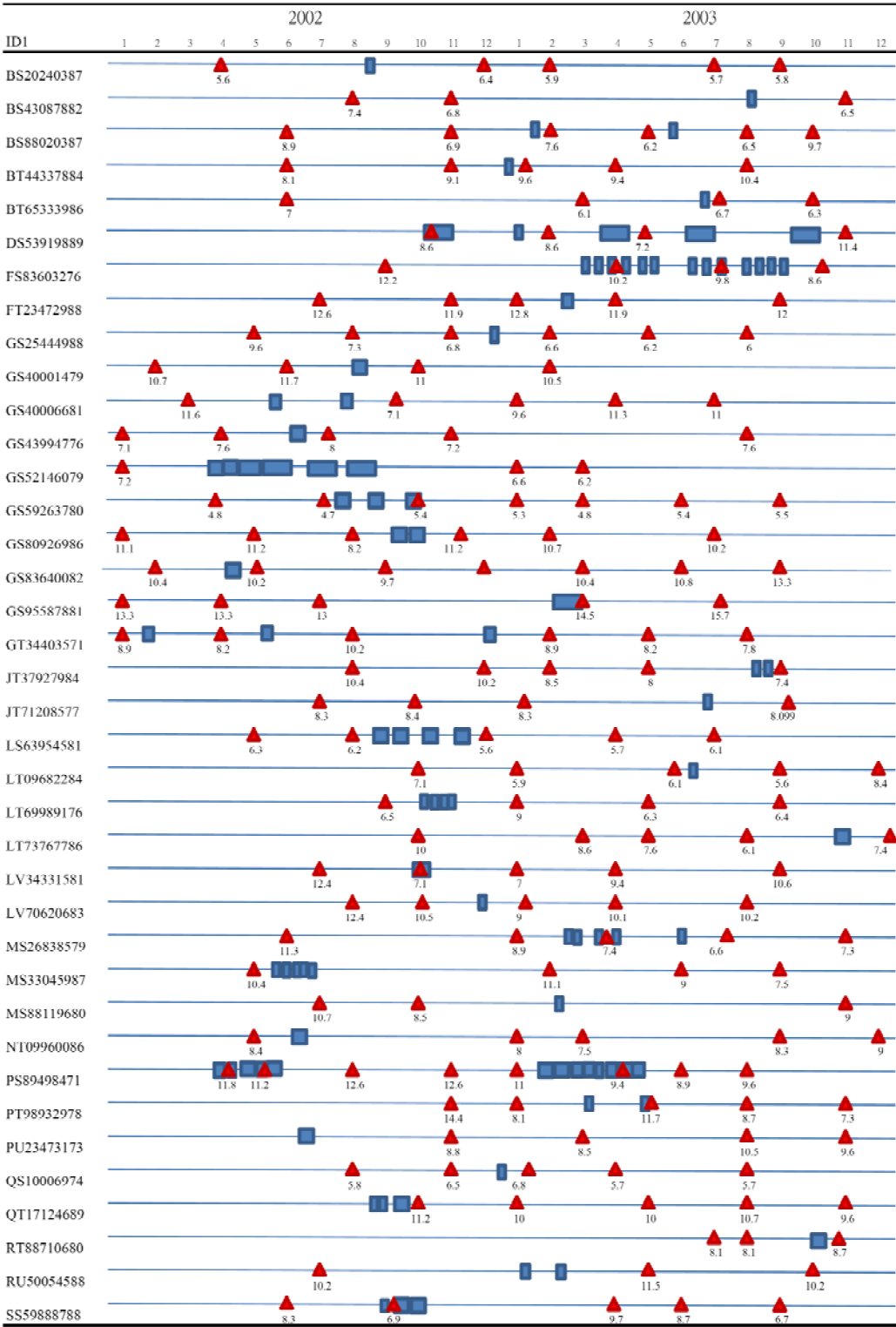

Supplement: Figure S2 — The duration of CYSKT administration and HbA1c measurement in diabetic patients. Rectangle represents the duration of CYSKT administration. Triangle represents the time of HbA1c measurement. The number below each triangle represents the HbA1c value (%). (PDF) [file pone.0104650.s002.pdf]

**Figure S3**

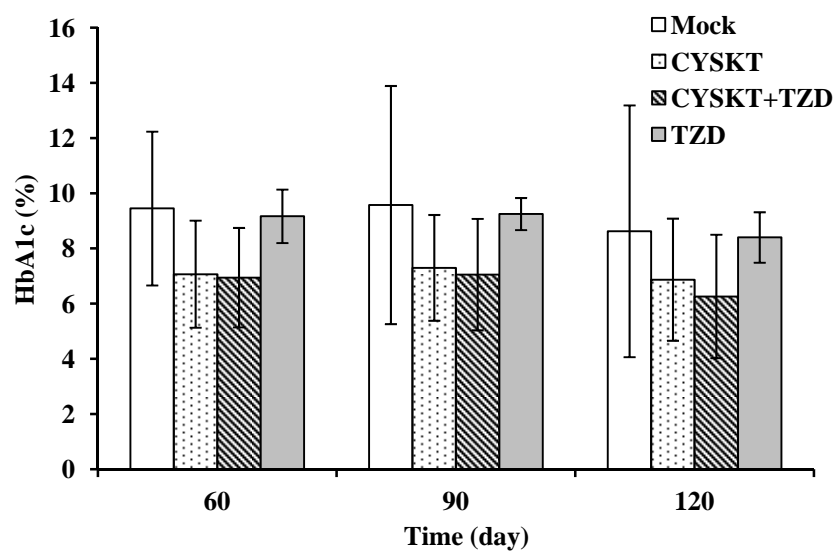

Supplement: Figure S3 — Effects of CYSKT on the HbA1c levels in type 2 diabetic mice. Type 2 diabetic mice were administered orally with 200 mg/kg CYSKT and/or 20 mg/kg TZD for 120 consecutive days. Sixty days after CYSKT administration, blood samples were collected every 30 days and measured for HbA1c levels. Values are mean ± SD (n = 5). (PDF) [file pone.0104650.s003.pdf]
