# Supplementary material for: A Novel Glycated Hemoglobin A1c-Lowering Traditional Chinese Medicinal Formula, Identified by Translational Medicine Study
Source: PLoS One. 2014 Aug 18;9(8):e104650. doi: 10.1371/journal.pone.0104650 (PMC4136774; doi:10.1371/journal.pone.0104650)
Supplement: Table S1 — The top ten commonly used Chinese medicinal formulae in diabetic patients. (PDF) [file pone.0104650.s004.pdf]

Table S1. The top ten commonly used Chinese medicinal formulae in diabetic patients.

Chinese medicinal formula	Ingredient	Total dosage (g) in each course of treatment	Total number of administration days in each course of treatment	Total dosage (g) of each patient	Total number of administration days of each patient
Liu-Wei-Di-Huang-Wan<br>六味地黃丸	Rehmannia (32%)<br>Cornus fruit (16%)<br>Chinese yam (16%)<br>Peony root (12%)<br>Poria (12%)<br>Alisma (12%)	57.09 ± 42.73	21.03 ± 22.02	187.52 ± 383.31	70.42 ± 150.36
Yu-Quan-Wan<br>玉泉丸	Trichosanthes root (16.7%)<br>Kudzu root (16.7%)<br>Ophiopogon tuber (11.1%)<br>Ginseng root (11.1%)<br>Poria (11.1%)<br>Plum (11.1%)<br>Licorice root (11.1%)<br>Astragalus root (11.1%)	58.03 ± 33.86	16.24 ± 14.73	272.23 ± 453.07	86.16 ± 128.41
Bai-Hu-Jia-Ren-Shen-Tang<br>白虎加參湯	Gypsum (45.7%)<br>Rice (22.9%)	47.22 ± 36.68	17.65 ± 16.78	153.32 ± 237.04	58.19 ± 93.65

	Common Anemarrhena Rhizome (17.1%)<br>Ginseng root (8.6%)<br>Licorice root (5.7%)				
Zhi-Bai-Di-Huang-Wan<br>知柏地黃丸	Common Anemarrhena Rhizome (22.2%)<br>Amur cork-tree bark (22.2%)<br>Rehmannia (17.7%)<br>Cornus fruit (8.9%)<br>Chinese yam (8.9%)<br>Peony root (6.7%)<br>Poria (6.7%)<br>Alisma (6.7%)	65.89 ± 56.29	19.16 ± 20.03	176.52 ± 360.17	51.34 ± 60.17
Ji-Sheng-Shen-Qi-Wan<br>濟生腎氣丸	Rehmannia (23.6%)<br>Poria (17.6%)<br>Cornus fruit (11.8%)<br>Chinese yam (11.8%)<br>Alisma (8.8%)<br>Peony root (8.8%)<br>Achyranthes root (5.9%)<br>Asiatic plantain (5.9%)	48.63 ± 29.02	15.28 ± 13.29	181.68 ± 271.60	58.72 ± 78.38

	Cinnamon twig (2.9%)<br>Aconite (2.9%)				
Qi-Ju-Di-Huang-Wan<br>杞菊地黃丸	Rehmannia (27.7%)<br>Cornus fruit (13.8%)<br>Chinese yam (13.8%)<br>Peony root (10.3%)<br>Poria (10.3%)<br>Alisma (10.3%)<br>Chinese wolfberry fruit (6.9%)<br>Chrysanthemum flower (6.9%)	57.16 ± 31.24	23.76 ± 17.97	168.57 ± 240.40	70.42 ± 116.04
Gan-Lu-Yin<br>甘露飲	Rehmannia (20%)<br>Ophiopogon tuber (10%)<br>Mature fruit of bitter orange (10%)<br>Licorice root (10%)<br>Capillaris (10%)<br>Loquat leaves (10%)<br>Dendrobium (10%)<br>Baical skullcap root (10%)<br>Asparagus tuber (10%)	42.33 ± 26.40	16.67 ± 17.44	116.68 ± 139.99	45.32 ± 48.41
Bai-Hu-Tang	Gypsum (50%)	47.29 ± 44.22	21.73 ± 16.52	128.21 ± 194.16	58.90 ± 90.86

白虎湯	Common Anemarrhena Rhizome (18.8%)<br>Licorice root (6.2%)<br>Rice (25%)				
Chu-Yeh-Shih-Kao-Tang<br>竹葉石膏湯	Bamboo leaves (5.1%)<br>Gypsum (41%)<br>Pinellia rhizome (10.3%)<br>Ginseng root (7.7%)<br>Licorice root (5.1%)<br>Rice (15.4%)<br>Ophiopogon tuber (15.4%)	46.63 ± 26.88	17.64 ± 11.69	122.72 ± 165.04	48.03 ± 65.56
Ba-Wei-Di-Huang-Wan<br>八味地黃丸	Rehmannia (29.7%)<br>Chinese yam (14.8%)<br>Cornus fruit (14.8%)<br>Alisma (11.1%)<br>Poria (11.1%)<br>Peony root (11.1%)<br>Cinnamon twig (3.7%)<br>Aconite (3.7%)	46.21 ± 26.52	21.35 ± 17.12	110.91 ± 132.26	50.71 ± 85.57
