# Supplementary material for: A Novel Glycated Hemoglobin A1c-Lowering Traditional Chinese Medicinal Formula, Identified by Translational Medicine Study
Source: PLoS One. 2014 Aug 18;9(8):e104650. doi: 10.1371/journal.pone.0104650 (PMC4136774; doi:10.1371/journal.pone.0104650)
Supplement: Table S2 — List of synthetic drugs used in patients who also administered CYSKT. (PDF) [file pone.0104650.s005.pdf]

Table S2. List of synthetic drugs used in patients who also administered CYSKT.

ID	Sex	Drugs				
		Metformin	Sulfonylurea	α-Glucosidase inhibitor	Thiazolidinedione	Insulin
BS20240387	F	+	+	−	−	−
BS43087882	F	+	+	+	+	−
BS88020387	F	+	+	+	+	+
BT44337884	F	+	+	−	+	−
BT65333986	F	−	+	−	−	−
DS53919889	F	+	−	−	−	−
FS83603276	M	+	+	+	+	−
FT23472988	F	+	+	−	−	+
GS25444988	F	+	+	−	−	−
GS40001479	M	+	+	−	−	+
GS40006681	F	+	+	−	+	+
GS43994776	M	+	−	−	−	−
GS52146079	M	+	+	−	−	−
GS59263780	F	+	+	−	−	−
GS80926986	F	+	+	+	+	−
GS83640082	F	+	+	+	+	+
GS95587881	F	+	+	−	+	+
GT34403571	M	+	+	−	+	−
JT37927984	F	+	+	−	−	−
JT71208577	M	+	+	−	−	−
LS63954581	F	+	+	−	−	−
LT09682284	F	+	+	−	−	−
LT69989176	M	+	+	−	−	−
LT73767786	F	+	+	−	+	−
LV34331581	F	+	+	+	+	−
LV70620683	F	+	+	−	+	−
MS26838579	M	+	+	−	−	−
MS33045987	F	+	+	+	+	−
MS88119680	F	−	+	−	+	−
NT09960086	F	+	+	+	+	−
PS89498471	M	−	−	+	+	−
PT98932978	M	+	+	−	+	−

PU23473173	M	+	+	−	+	−
QS10006974	M	−	+	−	−	−
QT17124689	F	+	+	−	+	−
RT88710680	F	−	−	−	−	+
RU50054588	F	+	+	+	−	−
SS59888788	F	−	+	−	−	−
