# Supplementary material for: A Novel Glycated Hemoglobin A1c-Lowering Traditional Chinese Medicinal Formula, Identified by Translational Medicine Study
Source: PLoS One. 2014 Aug 18;9(8):e104650. doi: 10.1371/journal.pone.0104650 (PMC4136774; doi:10.1371/journal.pone.0104650)
Supplement: Text S1 — Materials and Methods for Figure S1. (DOCX) [file pone.0104650.s006.docx]

**High-performance liquid chromatography (HPLC) analysis of ethanolic extract of CYSKT**

The ethanolic extract of CYSKT was freeze-dried and dissolved in ethanol to a final concentration of 10 mg/mL. Samples were filtered through a 0.22 μm membrane filter prior to HPLC injection. HPLC was performed on a Shimadzu HPLC system (Kyoto, Japan) equipped with Shimadzu LC-20AT pump, Shimadzu SIL-20 auto sampler, and Shimadzu SPD-M20A detector (wavelength: 190-800 nm). The HPLC profile was performed using a RP-18 column (SUPELCO, Discovery HS C_18_, 4.6 x 250 mm, 5 μm) at a flow rate of 1 mL/min and detected at 254 nm. The injection volume was 10 μL. The mobile phase was composed of solvent (A), 0.1% trifluoroacetic acid solution, and solvent (B), acetonitrile. The solvent gradient was as follows: 0-30 min from 5% B to 60% B; 30-50 min from 60% B to 90% B; 50-60 min from 90% B to 100% B. A series of standard solutions (glycyrrhizin) with different concentrations (5, 1, 0.5, 0.25, 0.125, 0.0625, and 0.0312 mg/mL) were prepared to calculate the concentration of glycyrrhizin in the ethanolic extract of CYSKT.
